# Leptospirosis Risk among Occupational Groups in Brazil, 2010–2015

**DOI:** 10.4269/ajtmh.21-0181

**Published:** 2023-07-03

**Authors:** Deise I. Galan, Maria Cristina Schneider, Amira A. Roess

**Affiliations:** ^1^ Department of Environmental and Occupational Health, The George Washington University, Washington, District of Columbia;; ^2^ Department of International Health, Georgetown University, Washington, District of Columbia;; ^3^ Institute of Studies in Collective Health, Federal University of Rio de Janeiro, Rio de Janeiro, Brazil;; ^4^ Department of Global and Community Health, George Mason University, Fairfax, Virginia

## Abstract

Leptospirosis is a zoonotic disease that is primarily transmitted through close contact with contaminated environments or infected animals. Brazil has the highest number of reported cases of leptospirosis in the Americas (approximately 4,000 annual cases). The purpose of this study is to identify the occupational groups with a higher risk of leptospirosis in Brazil from 2010 through 2015 among suspected cases reported to the national surveillance system. Confirmed and unconfirmed cases of leptospirosis with laboratory diagnosis, 20,193 and 59,034 respectively, were classified into 12 occupational groups. Confirmed cases were predominantly male (79.4%), between 25 and 59 years of age (68.3%), white (53.4%), illiterate or with incomplete primary education (51.1%), and participating in agricultural work (19.9%). After controlling for age, sex, race, and area of residency, the multivariate analysis identified that between confirmed and unconfirmed cases of leptospirosis reported to the Brazilian national surveillance system, five occupational groups are at higher risk for leptospirosis: garbage and recycling collectors (odds ratio [OR] = 4.10; 95% CI = 3.36–4.99); agricultural, forestry, and fishery workers (OR = 1.65; 95% CI = 1.49–1.84); prisoners (OR = 1.56; 95% CI = 1.04–2.35); building workers (OR = 1.36; 95% CI = 1.22–1.51); cleaners and mining workers (OR = 1.25; 95% CI = 1.07–1.45). This is the first nationwide study to examine leptospirosis risk by occupational group in Brazil using national surveillance data. Our results suggest that among suspected cases there was an increased risk among occupational groups with low income and low educational levels.

## INTRODUCTION

Leptospirosis is a zoonotic disease caused by spirochete bacterium of the genus *Leptospira*.^1^^,^^2^ Globally, it is estimated to cause 1.03 million cases and 58,900 deaths each year.^3^ Leptospirosis transmission occurs via direct or indirect exposure to the urine of infected animals, primarily through contaminated water or soil.^1^^,^^2^ Symptoms range in severity from a mild disease lasting about a week, characterized by fever, chills, headache, and myalgia, to a life-threatening illness causing severe multisystem complications such as jaundice, renal insufficiency, and pulmonary hemorrhage.^2^ The greatest burden of leptospirosis is in low-income tropical countries, especially among resource-poor populations with limited access to adequate sanitation, thus it is considered a neglected tropical disease.^3^^,^^4^

Brazil has the highest number of reported cases of leptospirosis in the Region of the Americas. Annually an average of 4,000 confirmed cases with a 10% case fatality are reported to the Ministry of Health (MOH) and the majority of cases are among young adults between the ages of 20 and 49 years old.^5^^,^^6^ Leptospirosis is prevalent across the country and occurs throughout the year, with increases and outbreaks primarily during the rainy season.

Occupation was recognized early on in the study of leptospirosis as an important risk factor for the disease. Miners were the first occupational risk group to be recognized, followed by fish workers in the early 1930s.^7^^,^^8^ Livestock farming is now considered one of the main occupational risk factors for leptospirosis worldwide.^1^^,^^9^^,^^10^ Due to the exposure risk among certain occupational groups and its potentially severe impact on the health of workers, leptospirosis has been recognized as an occupational disease by the International Labor Organization.^11^ A recently published systematic review of leptospirosis outbreaks from 1970 to 2012 found that 26% of outbreaks identified in their analysis occurred in an occupational setting, of which the majority (66%) were in rural areas.^12^

The risk of leptospirosis is greater in occupations with higher exposures to infected animals or contaminated environments. Direct contact with infected animals accounts for most infections in farmers, veterinarians, abattoir workers, meat inspectors, rodent control workers, and other occupations that require contact with animals.^1^^,^^9^^,^^10^^,^^13^ Indirect contact with water and soil contaminated with the urine of infected animals may expose sewer workers, miners, soldiers, septic tank cleaners, fish farmers, rice field workers, rubber tappers, and sugarcane harvesters.^14^^–^^18^

In Brazil, previous studies have shown the risk of leptospirosis among farmers, subsistence farm workers, dairy farmers, and abattoir workers.^19^^–^^22^ No previous study has compared the risk between occupational groups at the national level across several years in Brazil. The purpose of this study is to identify the occupational groups with a higher risk of leptospirosis in Brazil from 2010 through 2015 among suspected cases reported to the national surveillance system.

## METHODOLOGY

### Study design, data, and case definition.

A retrospective epidemiological study was carried out using leptospirosis surveillance data from the MOH of Brazil from 2010 through 2015 (6 years). The data were requested via the Citizen Information System and de-identified by the Brazilian MOH before sent to the researchers.^23^ Since 2000, leptospirosis was classified as a notifiable disease in Brazil through the Information System for Notifiable Diseases (acronym in Portuguese: SINAN). Healthcare professionals are required to complete a notification form for all suspected leptospirosis cases and register it on SINAN.^24^ The notification form collects information about the patient’s demographic characteristics, epidemiological history, occupation, clinical signs and symptoms, laboratory confirmation results, and final disease classification.^25^ Case investigation is conducted by the local authorities at the county level and then sent to health authorities at the state and national levels.^26^

A suspected case of leptospirosis is defined by the Brazilian MOH as an individual presenting with fever, headache, and myalgia with either one of the following criteria: 1) presence of suggestive epidemiological history in the last 30 days prior to the onset of symptoms, such as exposure to flood, sewage, or trash; activities involving occupational risk; epidemiological linkage with a laboratory confirmed case; living or working in a leptospirosis risk area; or 2) presence of one or more of the following signs or symptoms: jaundice, high levels of bilirubin, conjunctival suffusion, signs of acute renal failure, hemorrhagic phenomena.^24^

Suspected cases are confirmed by clinical-laboratory or clinical-epidemiological criteria. A laboratory confirmed case must have the presence of compatible clinical signs/symptoms and one or more of the following: reagent ELISA-IgM test with microagglutination test (MAT) seroconversion (two samples); 4-fold or greater increase in antibody titer by MAT (two samples) or one sample with titer equal to or greater than 800 by MAT; isolation of *Leptospira* from blood or positive polymerase chain reaction or immunohistochemistry for leptospirosis in suspected patients who eventually die.^24^ An epidemiologic confirmed case is defined as a suspected case who: 1) presents fever and changes in liver, renal, or vascular function; and 2) who has an epidemiological history (described in the definition of suspected cases above) but for some reason no laboratory confirmation (i.e., material for specific laboratory tests were not collected or the result of the serological test collected prior to the 7th day of illness was nonreactive).^24^ Unconfirmed leptospirosis cases are classified with and without laboratory diagnosis (ELISA-IgM or MAT) according to the Brazilian MOH case definition criteria provided in Supplemental File 1.

For the purpose of this study, only confirmed and unconfirmed cases of leptospirosis with laboratory diagnosis were included in our analysis. A flowchart illustrating the study design is provided in Supplemental File 2. The following variables were obtained from the surveillance data for each individual: age, sex, race, education, area of residency (urban/rural), state of residency, occupation, final case classification, and case classification criteria (laboratory or epidemiologic). For the purpose of this study, only individuals with age equal or greater to 14 years old were included in the analysis, since that is the official working age of apprentices in Brazil.^27^

### Classification of occupation.

A descriptive analysis was conducted to identify the occupational groups with the highest frequency in the dataset prior to aggregating the occupational groups. Subsequently, each occupation in the database was matched with its corresponding International Standard Classification of Occupations, ISCO-08 codes (Supplemental File 3).^28^ Some of the categories/subgroups that showed a high frequency in the descriptive analysis and that were relevant for leptospirosis transmission were categorized separately (e.g., garbage and recycling collectors). In total, 12 occupational groups were formed. The occupational reference group was those who were unemployed or retired since they may have less occupational exposure to leptospirosis, compared with other groups.

### Statistical analysis.

Descriptive and univariate analyses of the demographic characteristics (age, sex, race, and education), area of residence (urban/rural), and 12 occupational groups of confirmed and unconfirmed cases of leptospirosis were conducted. Multivariate analysis was performed using a logistic regression model to identify the occupational groups associated with leptospirosis. Odds ratio (OR) and 95% CI were calculated to estimate the association between independent factors and leptospirosis. Age, sex, race, and area of residence (urban or rural) were included in the final model because they are considered important risk factors in the literature. Education was excluded from the final model since it is a proxy for occupation. A likelihood-ratio test was performed to evaluate the goodness-of-fit of various models and a *P* value of 0.05 was considered for statistical significance. All statistical analyses were conducted using Stata 13.

### Maps.

For each occupational group identified in the final multivariate model with significant association with leptospirosis, the number of leptospirosis cases by area of residence (urban and rural) was mapped by state. Incidence rates were calculated by state based on the number of cases of leptospirosis during the study period per 100,000 population and included in the maps as reference. Population data was obtained from the latest Brazilian Census conducted in 2010 by the National Institute of Statistic of Brazil (Portuguese acronym: IBGE).^29^ Maps were constructed in ArcGIS versus 10.8.

## RESULTS

**Figure 1.**
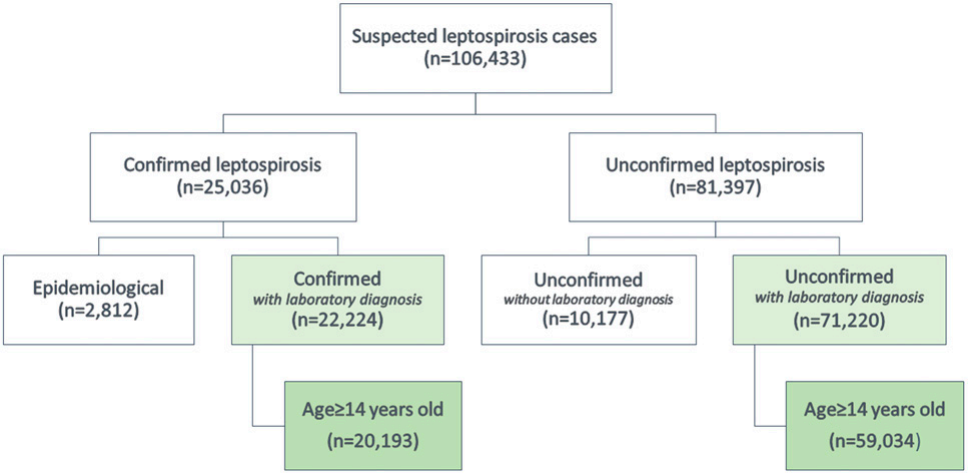
Flowchart of suspected cases with confirmed and unconfirmed diagnoses of leptospirosis with 14 years of age and older from national surveillance in Brazil, 2010–2015.

A total of 106,433 suspected cases of leptospirosis were reported to the Brazilian MOH from 2010 through 2015, of which 25,036 and 81,397 had respectively, a confirmed and unconfirmed leptospirosis diagnosis. Among these, 22,224 cases were confirmed with laboratory diagnosis and 71,220 were unconfirmed with laboratory diagnosis. According to the methodology, only individuals with age equal or greater to 14 years old were included in the analysis, 20,193 confirmed and 59,034 unconfirmed cases of leptospirosis (Figure 1).

Confirmed cases were predominantly males (79.4%), between 25 and 59 years of age (68.3%), white (53.4%), illiterate or with incomplete primary education (51.1%; Table 1). Most confirmed cases lived in urban areas (83.1%) and were from the south and southeast regions. Univariate analysis showed that among suspected cases of leptospirosis reported to the Brazilian national surveillance system the risk for acquiring leptospirosis is greatest in males (OR = 2.18), between 40 and 59 years (OR = 1.28), and from mixed race background (OR = 1.19). Leptospirosis risk was higher among those who were less educated, lived in rural areas (OR = 1.60) and were from the northeast region of Brazil (OR = 2.96).

**Table 1:** Demographic characteristics of cases with confirmed and unconfirmed diagnoses of leptospirosis among suspected leptospirosis cases reported to the national surveillance in Brazil, 2010–2015

Characteristic	Confirmed diagnosis (*N* = 20,193), *n* (%)^d^	Unconfirmed diagnosis (*N* = 59,034), *n* (%)§	Univariate OR (95% CI)
Age*			
14–24	4,511 (22.3)	13,365 (22.6)	1.18 (1.11–1.25)
25–39	6,703 (33.2)	19,791 (33.5)	1.18 (1.11–1.25)
40–59	7,079 (35.1)	19,257 (32.6)	1.28 (1.21–1.36)
≥ 60	1,900 (9.4)	6,621 (11.2)	Reference
Sex†			
Male	16,028 (79.4)	37,714 (63.9)	2.18 (2.09–2.26)
Female	4,165 (20.6)	21,318 (36.1)	Reference
Race†			
Black	1,065 (5.9)	3,218 (6.1)	1.02 (0.95–1.10)
Mixed	7,223 (39.9)	18,814 (35.8)	1.19 (1.15–1.23)
Others‡	164 (0.9)	622 (1.2)	0.82 (0.69–0.97)
White	9,673 (53.4)	29,918 (56.9)	Reference
Education†			
Illiterate/incomplete primary education	6,622 (51.1)	15,753 (40.9)	2.63 (2.36–2.92)
Primary education	3,016 (23.3)	8,923 (23.1)	2.11 (1.89–2.36)
Secondary education	2,882 (22.3)	11,217 (29.1)	1.60 (1.44–1.79)
Higher education	428 (3.3)	2,673 (6.9)	Reference
Area of residence†			
Rural	3,249 (16.9)	6,422 (11.3)	1.60 (1.53–1.67)
Urban	15,925 (83.1)	50,319 (88.7)	Reference

OR = odds ratio.

* Age ≥ 14 years old.

† Total numbers may not add up due to missing values.

‡ East Asian and Indigenous.

§ Percentages are reported as row percentages.

**Figure 2.**
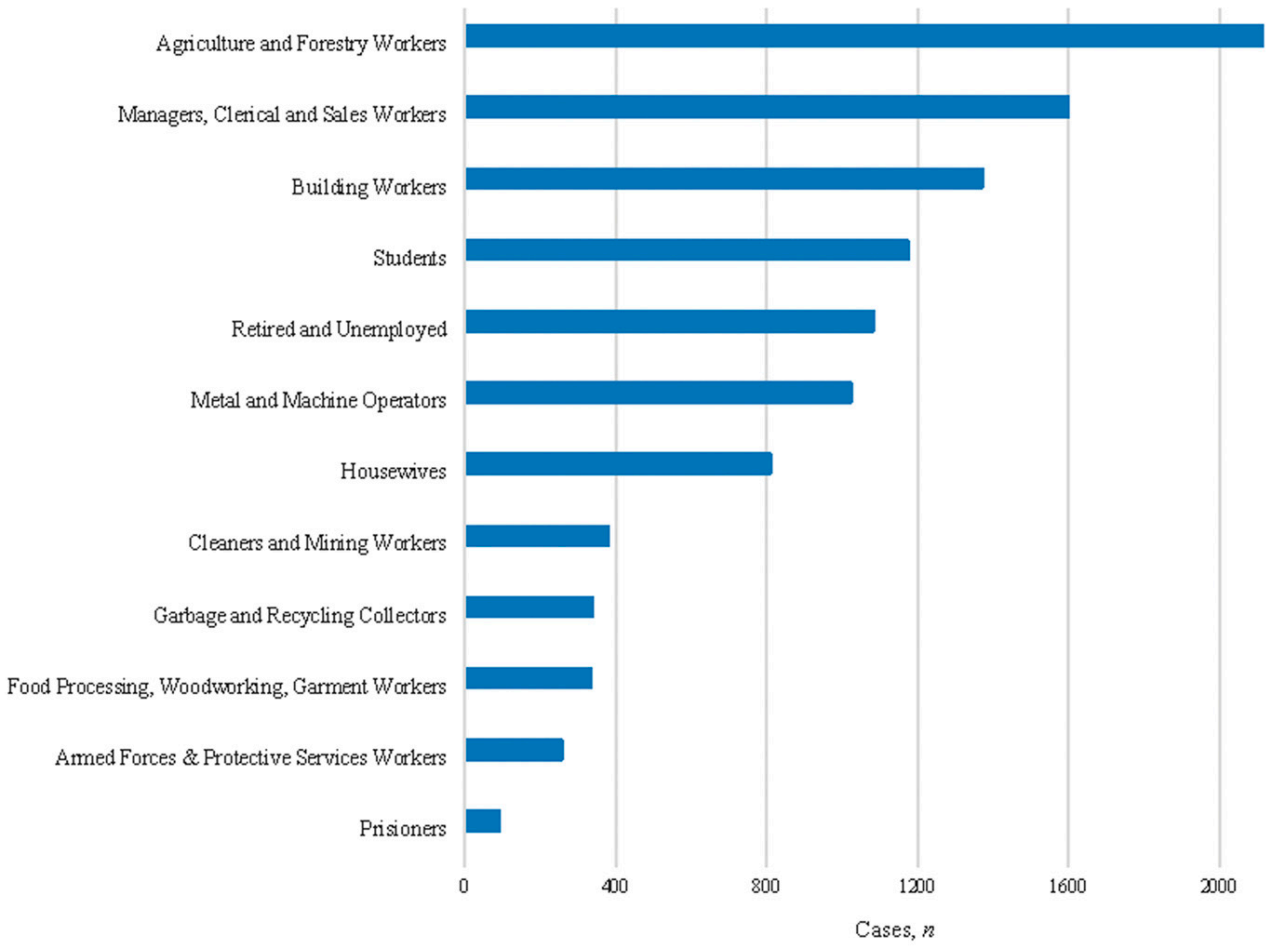
Occupational groups of cases with confirmed diagnosis of leptospirosis in Brazil, 2010–2015.

Among confirmed cases with occupation information, approximately 20% were agricultural, forestry, and fishery workers, followed by managers, professionals, clerical, and sales workers (15.2%), and building workers (13.0%; Figure 2, Table 2). In the univariate analysis, garbage and recycling collectors, prisoners and agricultural, forestry, and fishery workers were associated with leptospirosis compared with retired and unemployed individuals.

**Table 2:** Occupational groups of cases with confirmed and unconfirmed diagnoses of leptospirosis among suspected leptospirosis cases reported to the national surveillance system in Brazil, 2010–2015

Occupation	ISCO codes	Confirmed diagnosis (*N* = 10,457), *n* (%)	Unconfirmed diagnosis (*N* = 29,599), *n* (%)	Unadjusted OR
Agricultural and forestry workers	6, 92, 2132, 2250, 3142, 3240, 5164, 7233, 8341, 9332, 9333	2,083 (19.9)	3,196 (10.8)	2.09 (1.92–2.29)
Armed forces & protective services workers	0, 54, 3355	256 (2.5)	614 (2.1)	1.33 (1.13–1.56)
Building workers	71	1,358 (13.0)	2,468 (8.3)	1.76 (1.60–1.93)
Cleaners and mining workers	91, 93, 5151, 9613	378 (3.6)	1,122 (3.8)	1.07 (0.94–1.23)
Food processing, woodworking, garment workers	75, 815, 816, 817	329 (3.2)	961 (3.3)	1.09 (0.95–1.26)
Garbage and recycling collectors	9611	333 (3.2)	240 (0.8)	4.44 (3.71–5.31)
Housewives	N/A	796 (7.6)	3,831 (13.0)	0.66 (0.60–0.74)
Machine operators, metal, and electrical workers	72, 73, 74, 81, 82, 83	1,029 (9.8)	3,115 (10.5)	1.04 (0.95–1.15)
Managers, professionals, clerical, and sales workers	1, 2, 3, 4, 5	1,585 (15.2)	6,847 (23.1)	0.74 (0.68–0.81)
Prisoners	N/A	88 (0.8)	87 (0.3)	3.23 (2.39–4.38)
Students	N/A	1,148 (11.0)	3,685 (12.5)	1.00 (0.91–1.10)
Retired and unemployed	N/A	1,074 (10.3)	3,433 (11.6)	Reference

OR = odds ratio.

In the multivariate analysis (Table 3), after controlling for age, sex, race, and area of residency, five occupational groups showed a significant higher risk for leptospirosis compared with retired and unemployed individuals: garbage and recycling collectors (OR = 4.10; 95% CI = 3.36–4.99); agricultural, forestry, and fishery workers (OR = 1.65; 95% CI = 1.49–1.84); prisoners (OR = 1.56; 95% CI = 1.04–2.35); building workers (OR = 1.36; 95% CI = 1.22–1.51); cleaners and mining workers (OR = 1.25; 95% CI = 1.07–1.45).

**Table 3:** Multivariable analysis of leptospirosis among suspect cases reported to the national surveillance system in Brazil, 2010–2015

Variables	Multivariate OR (95% CI)
Occupational groups	
Agricultural, forestry, and fishery workers	1.65 (1.49–1.84)
Armed forces & protective services workers	0.99 (0.83–1.19)
Building workers	1.36 (1.22–1.51)
Cleaners and mining workers	1.25 (1.07–1.45)
Food processing, woodworking, garment workers	1.08 (0.92–1.26)
Garbage and recycling collectors	4.10 (3.36–4.99)
Housewives	1.09 (0.96–1.23)
Machine operators, metal, and electrical workers	0.85 (0.76–0.95)
Managers, professionals, clerical, and sales workers	0.78 (0.71–0.87)
Prisoners	1.56 (1.04–2.35)
Students	1.02 (0.90–1.15)
Retired and unemployed	Reference
Age	
14–24	1.16 (1.04–1.29)
25–39	1.18 (1.07–1.30)
40–59	1.27 (1.16–1.39)
≥ 60	Reference
Sex	
Male	2.26 (2.11–2.43)
Female	Reference
Race	
Black	0.95 (0.86–1.06)
Mixed*	1.20 (1.14–1.27)
Others	0.80 (0.62–1.02)
White	Reference
Area of residency	
Rural	1.25 (1.16–1.35)
Urban	Reference

OR = odds ratio.

* East Asian and Indigenous.

**Figure 3.**
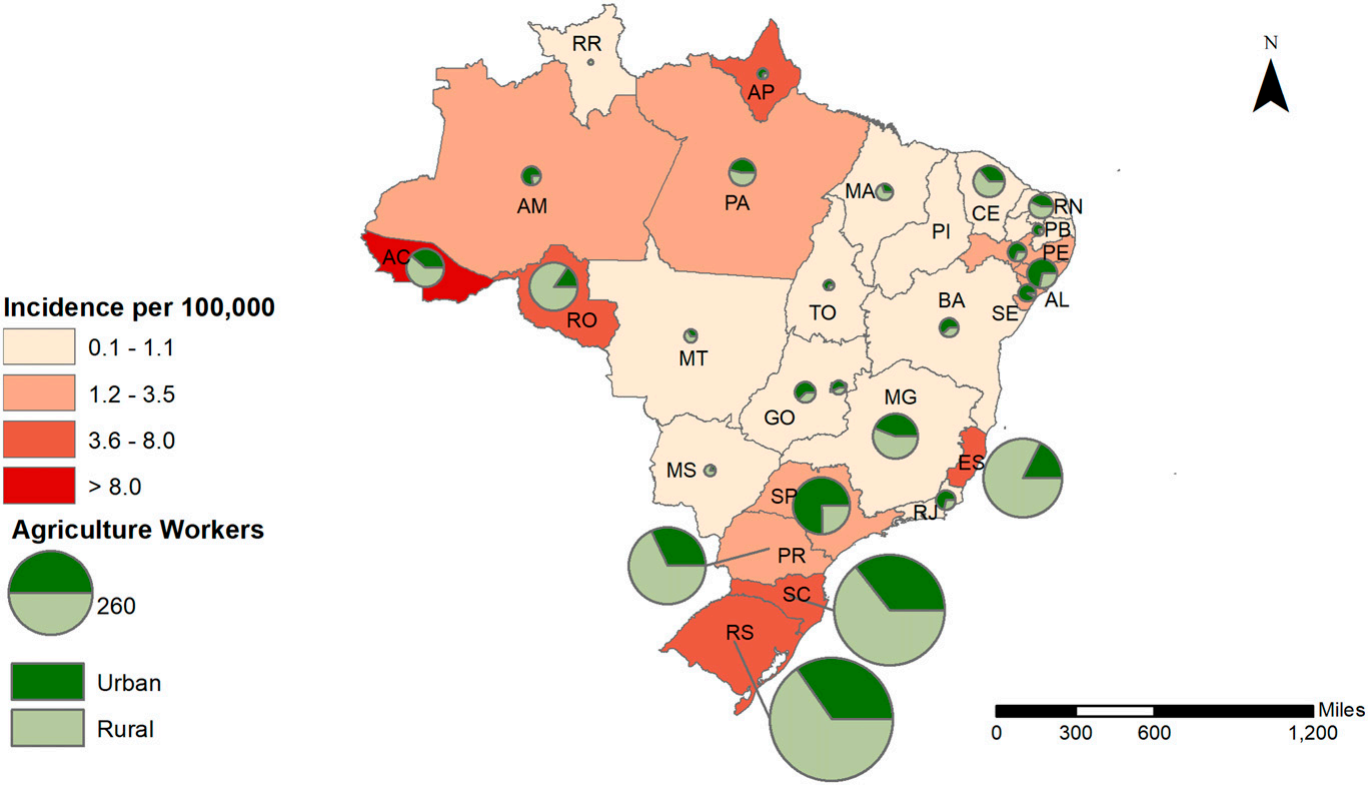
Incidence of leptospirosis and agricultural, forestry, and fishery workers in urban and rural areas of Brazil, 2010–2015.

**Figure 4.**
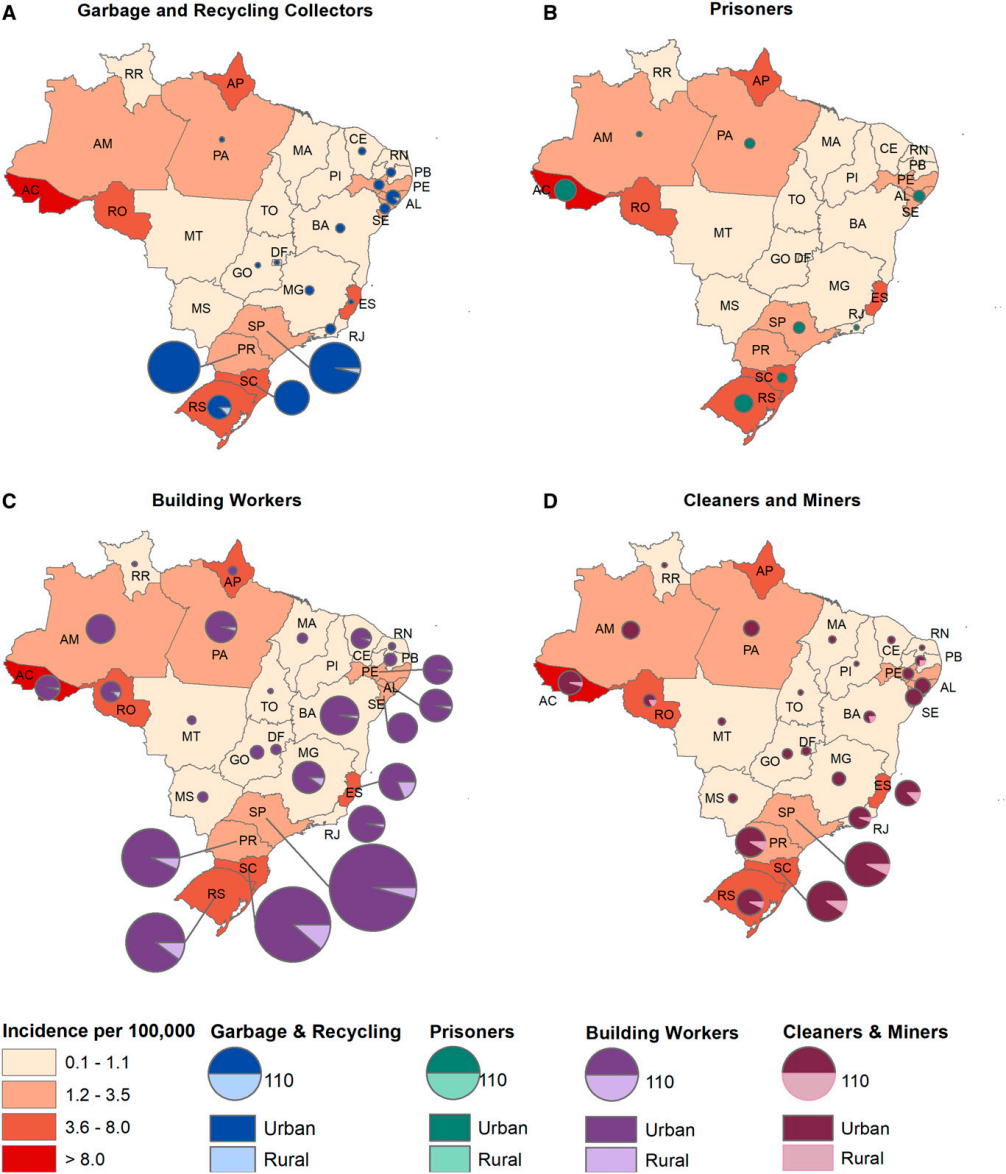
Incidence of leptospirosis and major occupational groups in urban and rural areas of Brazil. (**A**) Garbage and recycling collectors, (**B**) prisoners, (**C**) building workers, and (**D**) cleaners and miners, 2010–2015.

Maps were created of the five occupational groups associated with higher risk of leptospirosis to examine differences by urban/rural location within Brazil’s five regions. Conformed cases of leptospirosis among agricultural, forestry, and fishery workers were predominately located in the south and southeast regions: rural areas of the states of Parana, Santa Catarina, Rio Grande do Sul, and Espirito Santo; and urban areas of the state of São Paulo (Figure 3). Confirmed cases among garbage and recycling collectors were predominantly urban and located in the state of São Paulo and southern states. Prisoners with leptospirosis were all located in urban areas and reported in 8 out of 27 states (including the Federal District), with a larger number in the state of Acre and Rio Grande do Sul. Confirmed cases among building workers were identified throughout the country, although predominantly in urban areas of São Paulo and the south region. Smaller number of confirmed cases in cleaners and miners were also reported across Brazilian urban areas (Figure 4).

## DISCUSSION

This is the first nationwide study to examine leptospirosis risk by occupational group in Brazil. Because of the zoonotic nature of leptospirosis transmission, risk is exacerbated among occupational groups with direct contact to infected animals or their urine. This study identified that among suspected leptospirosis cases reported to the national surveillance system, three occupational groups were at a higher risk of leptospirosis: garbage and recycling collectors, prisoners and building workers, while ratifying the risk among agricultural workers, cleaners, and miners. The results of this study corroborates that leptospirosis is a poverty-related disease, affecting mostly occupational groups with low income and low educational levels.

Our study found that among suspected leptospirosis cases, garbage and recycling collectors have the highest risk for leptospirosis compared with other occupational groups in Brazil. Garbage and recycling collectors play a fundamental role in Brazil’s selective waste collection and recycling industry by contributing to the reduction of waste, extending the life cycle of products, decreasing the operating cost and volume of landfills, and reducing the consumption of raw materials, while passively helping the environment.^30^^,^^31^ Concentrated mainly in densely populated urban areas with higher per-capita GDP of the south and southeast regions, and formally recognized as a professional category in 2002 under the Brazilian Classification of Occupation, garbage and recycling collectors are responsible for the collection, selection, and sales of recyclable materials, working independently on the streets or as part of cooperatives in recycling screening facilities.^32^^,^^33^

The demographic and socioeconomic profile of garbage and recycling collectors highlight their other risk factors for leptospirosis. Based on the most recent national census conducted in 2010, garbage and recycling collectors are predominately males, illiterate and with a medium age of 39 years old.^34^ With over half of garbage and recycling collectors being part of the informal sector in Brazil, this group suffers from low wages without benefits, precarious working conditions and exposure to several risk factors that puts this occupational group at higher vulnerability to working conditions that favor the transmission of leptospirosis and other zoonotic and infectious diseases, without adequate personal protective equipment (PPE) and education about the risk of contamination.^33^^,^^34^ Although exposure to trash has been reported in the literature as a risk factor for leptospirosis, few studies have focused on the occupational risk among garbage collectors, focusing mainly on municipal service workers with activities on sewage, town cleaning, and trash disposal, not specifically on recycling collectors.^35^^–^^39^ The results of this study bring to light the risk of leptospirosis among those working in the collection and screening of recycling materials, especially in urban areas of Brazil.

Prisoners were found to be the third occupational group with the highest risk for leptospirosis in Brazil among suspected cases reported to the national surveillance system. The Brazilian prison system has the fourth largest incarcerated population in the World and is a complex bureaucracy.^40^ With more than 607,000 people in custody, mostly living in overcrowded, humid, precarious and dark environments, its population has grown 575% from the early 1990s to 2014 and is composed of 75% of young and black people, 67% poorly educated and 41% are pretrial detainees.^40^ Prison environments coupled with detainee’s poor nutrition, sedentary lifestyle and widespread use of drugs in custody, accounts for high prevalence of communicable and noncommunicable diseases.^40^

Due to its confined settings, usually with poor environmental conditions that may expose inmates to contaminated water and rodent infestation, prisons have many of the risk factors for leptospirosis. A recent study about health morbidity in Brazilian prisons also using information from the National Information System for Notifiable Diseases obtained from 2007 to 2014, demonstrated the risk of infectious diseases for those who are incarcerated.^41^ There was an increase in the reporting of notifiable diseases across the country, with tuberculosis (64.4%), dengue (9.1%), AIDS (9.0%), and viral hepatitis (5.9%) among the most frequently reported diseases during the study period.^41^ Cases of leptospirosis among prisoners have been reported sporadically in the literature from a prison farm in the Philippines, a high security prison in South Africa and another from a social rehabilitation facility in Ecuador.^42^^–^^44^ Our study found the highest cases of leptospirosis among prisoners in urban areas of the state of Acre (north region) and Rio Grande do Sul (south region). Acre is the state with the fourth highest rate of prisoners per 100,000 inhabitants and the highest urban incidence rate of leptospirosis in Brazil.^6^^,^^40^ Rio Grande do Sul do not present high rates of prisoners and cases of leptospirosis in urban areas compares with the average of Brazil.^6^ Future studies in Brazil should investigate why this state has one of the highest number of cases of leptospirosis among detainees.

Our study identified building workers as another previously unrecognized occupational risk group for leptospirosis in Brazil among suspected cases reported to the national surveillance system. Construction workers are constantly exposed to water and soil that can be contaminated with the urine of infected animals, particularly those workers undertaking the first stages of the construction project and responsible for groundwork. Previous studies have shown an association between exposure to stagnant water and mud, and an increased risk for leptospirosis.^2^^,^^45^^,^^46^ A recently published systematic review of *Leptospira* in water and soil has shown that there is strong evidence that the bacteria can survive and remain infectious for a prolonged time in the environment.^47^ The risk of leptospirosis among building workers has not been extensively reported in the literature. One hospital-based seroprevalence study in India identified building workers to have a significantly greater odds of leptospirosis among urban cases compared with other occupations.^48^ Our study showed that building workers in urban areas of the south and southeast regions are also at higher risk of contracting leptospirosis. Future studies should be conducted to better understand the risk among this occupational group and identify more targeted prevention activities.

Agricultural workers are the most well-known occupational risk group for leptospirosis and have been studied across the globe, with most having either direct or indirect contact with animals. Rice farmers and other crop cultivators, including sugarcane, taro, banana, and oil palm, have been identified as a high-risk group for leptospirosis due to working environments that favors the transmission of *Leptospira*, including presence of rodent populations in and around the fields they work in, exposure to contaminated water and wet environments, presence of skin wounds, and lack of use of PPE.^14^^–^^18^^,^^49^ The use of vaccines for this high-risk group has been reported in Cuba, Japan, Korea, and France.^26^^,^^50^^–^^54^ In Japan, the number of cases of leptospirosis decreased dramatically since the 1960s, when mechanization of agriculture started, and at the same time, an inactivated vaccine against *Leptospira* was introduced to this population, coupled with the use of PPE.^50^ In other countries, however, where rice cultivation is conducted mostly manually with directly contact with water for prolonged periods of time, workers continue to be exposed to environments favorable for leptospirosis transmission and leptospirosis continues to be endemic.^15^^,^^16^^,^^55^

Animal workers, included among the group of agricultural workers in this study, have been identified as one of the main presumptive exposure factors reported by leptospirosis cases in rural areas of Brazil.^6^ Livestock farmers are often exposed directly to animal urine or may become infected indirectly by walking barefoot where animals have urinated.^45^^,^^56^ In many countries, domesticated animals, particularly swine and cattle, are important reservoirs for *Leptospira* and are a frequent source of infection for subsistence livestock farmers.^57^^–^^59^ In the northern and southern regions of Brazil, where leptospirosis cases among agricultural workers were found to be the highest in this study, other studies have also reported greater risk of leptospirosis in dairy and subsistence farmers there.^19^^–^^21^ The state of Rio Grande do Sul (south region), identified in our study with the highest number of leptospirosis cases among agricultural workers in rural areas, produces 76% of all rice in Brazil, has approximately one-third of its agricultural area based on family farming, and is one of the largest producers of swine and bovine in the country.^60^ Previous studies that analyzed the environmental and socioeconomic drivers for leptospirosis in that state demonstrated the presence of higher number of cases of leptospirosis in intensive agricultural production areas, including rice plantations.^61^^,^^62^

Miners were the first occupational group to be identified as high-risk for leptospirosis in the 1920s and are well-described in the literature.^7^^,^^8^^,^^63^^,^^64^ Due to the nature of their work, miners are susceptible to acquiring skins abrasions, which can facilitate the exposure to *Leptospira* in contaminated environments. A recent seroprevalence study conducted among miners in India determined that other occupational exposures also put this group at high-risk for leptospirosis, including exposure to water bodies on the way to the mines, water-logged environments, rat infestations and cattle rearing surrounding the mine.^64^ In Brazil, similar environmental exposures may occur among miners, but further studies are needed to better understand the occupational risk among this group in Brazil, especially in the state of São Paulo (southeast region) that has the highest number of cases.

Previous studies have shown that rodents play a major role in the transmission cycle of *Leptospira* in both urban and rural areas, especially among certain occupational groups.^2^^,^^45^^,^^46^ Agricultural workers mostly related to grain production, such as rice farmers, are in close contact with environments contaminated with the urine of infected rodents.^65^^–^^68^ In urban areas, garbage and recycling collectors are in close contact with garbage and wet environments that are prone to rodent infestations.^33^^,^^34^ A previous study conducted using Brazil’s national surveillance data showed that among leptospirosis cases, exposure to places with signs of rodents was the most often identified exposure factor in both urban and rural areas.^6^ Rodent control activities and changes to the environment to reduce rodent populations (i.e., through improved sanitation and waste collection) are successful public health strategies against leptospirosis, which should be taken into consideration.^46^^,^^69^

This study has some limitations. When assessing occupational risk, this study only focused on cases suspected of having leptospirosis that were reported to the national surveillance system, and compared confirmed cases to unconfirmed cases. We did not compare confirmed cases to the general population and hence can only report on risk of leptospirosis among persons reported to the national surveillance system who were suspected of having leptospirosis rather than risk among the general population. In our study, confirmed and unconfirmed leptospirosis cases were obtained from the same suspected leptospirosis database following the MOH guidelines for case definition criteria, which includes clinical-epidemiological and clinical-laboratory confirmation, and have a final classification as confirmed or unconfirmed diagnosis of leptospirosis. To avoid misclassification, only confirmed and unconfirmed cases of leptospirosis with laboratory diagnoses were included in our analysis. To assess the occupational risk of leptospirosis in the general population, future studies need to be conducted comparing confirmed cases to the underlying occupational rates in the general population.

Although Brazil has a robust national surveillance system, it relies on passive case ascertainment that requires patients reaching the healthcare system and being appropriately diagnosed and then being reported to the national authorities. The true number of leptospirosis cases in Brazil may be actually larger than reported in this study. This may be due to the fact that leptospirosis often presents with mostly mild and nonspecific symptoms, thus patients may not seek care and those who do reach the healthcare system may be misdiagnosed.^1^^,^^2^ Although the authors recognize the limitations of using the SINAN, several researchers have been using this database in scientific studies.^6^^,^^41^^,^^61^^,^^70^^–^^73^ Another limitation is that our study population had more males compared with the general population, this likely due to the fact that leptospirosis is most commonly suspected among males by healthcare professionals.

The classification by occupation has also some limitations. The notification form used to collect the patient’s information is completed by the healthcare professional and occupation is not a required field. Consequently, a large number of cases in our database did not have an occupation listed and were not included in our multivariate analysis. For the purpose of this study, researchers carefully aggregated occupations based on 12 main groups. To avoid misclassification during this process, a second researcher verified the categorization process. Although this study has limitations, the large number of cases with occupation information included in the study is robust and the largest from Brazil.

Our results corroborate previous reports from the MOH about the demographic characteristics and geographic distribution of leptospirosis cases in Brazil, predominantly among prime working-age white males (ages 24–59 years) living in urban areas.^74^ In our study, approximately 80% of confirmed diagnoses of leptospirosis were males in the 25- to 59-year age groups, corroborating previous studies that leptospirosis occur predominately among working-age males.^3^ This study highlights the importance of understanding and identifying the local and regional high-risk groups for leptospirosis across the country, given Brazil’s large territorial extension with diverse climatic and socioeconomic characteristics. Our study identified different occupational groups for leptospirosis in both urban and rural areas of Brazil with possible different exposure risk factors, and subsequently unique prevention strategies. Previous studies have also shown an association between lower educational levels and higher risk of acquiring leptospirosis.^75^ Similarly, our study identified that more than half of leptospirosis cases in Brazil are among those who report that they are illiterate or have incomplete primary education, demonstrating that this neglected zoonotic disease affects the most poor and marginalized populations.

Nevertheless, this study suggests that leptospirosis is a poverty-related disease affecting several occupational groups with low income such as agricultural/forestry workers, miners, and building workers, and garbage and recycling collectors. Another important finding was that among suspected cases there was an increased risk of leptospirosis among prisoners highlighting the need to address the conditions in Brazil’s prisons. Among confirmed and unconfirmed cases of leptospirosis reported to the Brazilian national surveillance system, all the occupational groups identified in the multivariate analysis with a higher risk for leptospirosis have direct or indirect exposure to environments that facilitate the transmission of leptospirosis, such as contact with animals, contaminated water, soil, and garbage. Previous studies conducted in Brazil demonstrated that although the number of leptospirosis cases in rural areas are lower compared with urban environments, the risk of leptospirosis is much higher in rural settings, which may be due to greater environmental and occupational exposure in these settings.^6^^,^^61^

The results of this study suggest that a strong collaboration among Health and Agriculture sectors are needed. Prevention and control strategies must involve multi-sectorial stakeholders from government, local associations, employers, and others under the One Health approach, a collaborative and multidisciplinary effort that views how animals, humans, and their shared environment are affected by the socioeconomic interest of humans.^76^^,^^77^ Intervention activities for leptospirosis prevention and control in Brazil should target these occupational groups, focusing on education and use of PPE, while considering the provision of PPE considering that many of the affected populations are low income and cannot afford to purchase boots, gloves, and other protective equipment. Due to Brazil’s tropical and subtropical climate, compliance regarding the use of PPE among workers is low, especially during long shifts and during the summer and rainy seasons, the periods with the highest number of cases. The most important prevention tool for high-risk occupational groups is vaccination; however, human vaccine is currently not available in the majority of endemic countries. Until the prevention of leptospirosis in high-risk groups is achieved, this neglected disease will continue to affect the most vulnerable populations in developing countries. The complex nature of the transmission of leptospirosis, gap of practical tools and fact that it is a silent epidemic disease that suffers from unawares, remains a major challenge for the scientific community and governments to address.

## Supplemental Materials


Supplemental materials

Supplemental materials
