# Supplementary material for: Leptospirosis Risk among Occupational Groups in Brazil, 2010–2015
Source: Am J Trop Med Hyg. 2023 Jul 3;109(2):376–86. doi: 10.4269/ajtmh.21-0181 (PMC10397431; doi:10.4269/ajtmh.21-0181)
Supplement: Supplementary file 1 [file tpmd210181.SD1.pdf]

Leptospirosis Case Definition

Translated from the Ministry of Health of Brazil. 2017 Health Surveillance Guide[1]

A. Suspected Case

An individual presenting with fever, headache and myalgia with either one of criteria listed below.

Criteria 1

Presence of suggestive epidemiological history in the 30 days prior to the date of onset of symptoms, such as:

- exposure to flood, mud or water reservoirs;
- exposure to sewage, pits, garbage and rubble;
- activities involving occupational risk, such as garbage collection and material for recycling, cleaning streams, working in water or sewage, handling animals, agriculture in flooded areas;
- epidemiological link to a case confirmed by laboratory criteria;
- living or working in areas at risk for leptospirosis.

Criteria 2

Presence of one or more of the following signs or symptoms:

- jaundice;
- high levels of bilirubin;
- conjunctival suffusion;

- hemorrhagic phenomena;
- signs of acute renal failure.

B. Confirmed Case

Criteria clinical- epidemiologic

All suspected cases that present fever and changes in liver, renal or vascular functions, associated with an epidemiological history (described in the definition of a suspected case) and that collection of material for specific laboratory tests were not possible, or these have been non-reagent with single sample collected before the 7th day of illness.

A **negative** (non-reactive) result of any specific serological test for leptospirosis (ELISA-IgM, microagglutination), with blood sample collected before the 7th day of onset of symptoms, does not discard a suspected case. Another blood sample should be collected, after the 7th day of onset of symptoms, to assist in the interpretation of the diagnosis, as previously mentioned.

Criteria clinical-laboratory

Suspected case associated with one or more of the following laboratory test results:

- reagent enzyme-linked immunosorbent assay (ELISA-IgM), plus microagglutination test (MAT) seroconversion with two samples, with a non-reagent first sample (acute phase) and a second sample (14 days after the onset of symptoms with a maximum of up to 60 days) with a titer greater than or equal to 200;

- four-fold or greater increase in antibody titer by MAT between two blood samples collected with an interval of approximately 14 days after the onset of symptoms (maximum 60 days) between them;
- when two or more samples are not available, a titer greater than or equal to 800 in the MAT confirms the diagnosis
- isolation of *Leptospira* from blood.

C. Discarded Case *(in our study referred as unconfirmed with laboratory diagnosis)*

- ELISA IgM test non-reagent from blood sample collected after the 7th day of onset of symptoms. In patients from rural areas, the clinician should also consider clinical history and epidemiological history to close the case.
- Two non-reagent microagglutination reactions (or reagent with no seroconversion neither a 4-fold or greater increase in titers), with blood samples collected from the first patient's visit and with an interval of 2 to 3 weeks between them.

Reference

1. Ministry of Health of Brazil, 2017. Guia de Vigilância em Saúde. Brasilia: Ministério da Saúde.

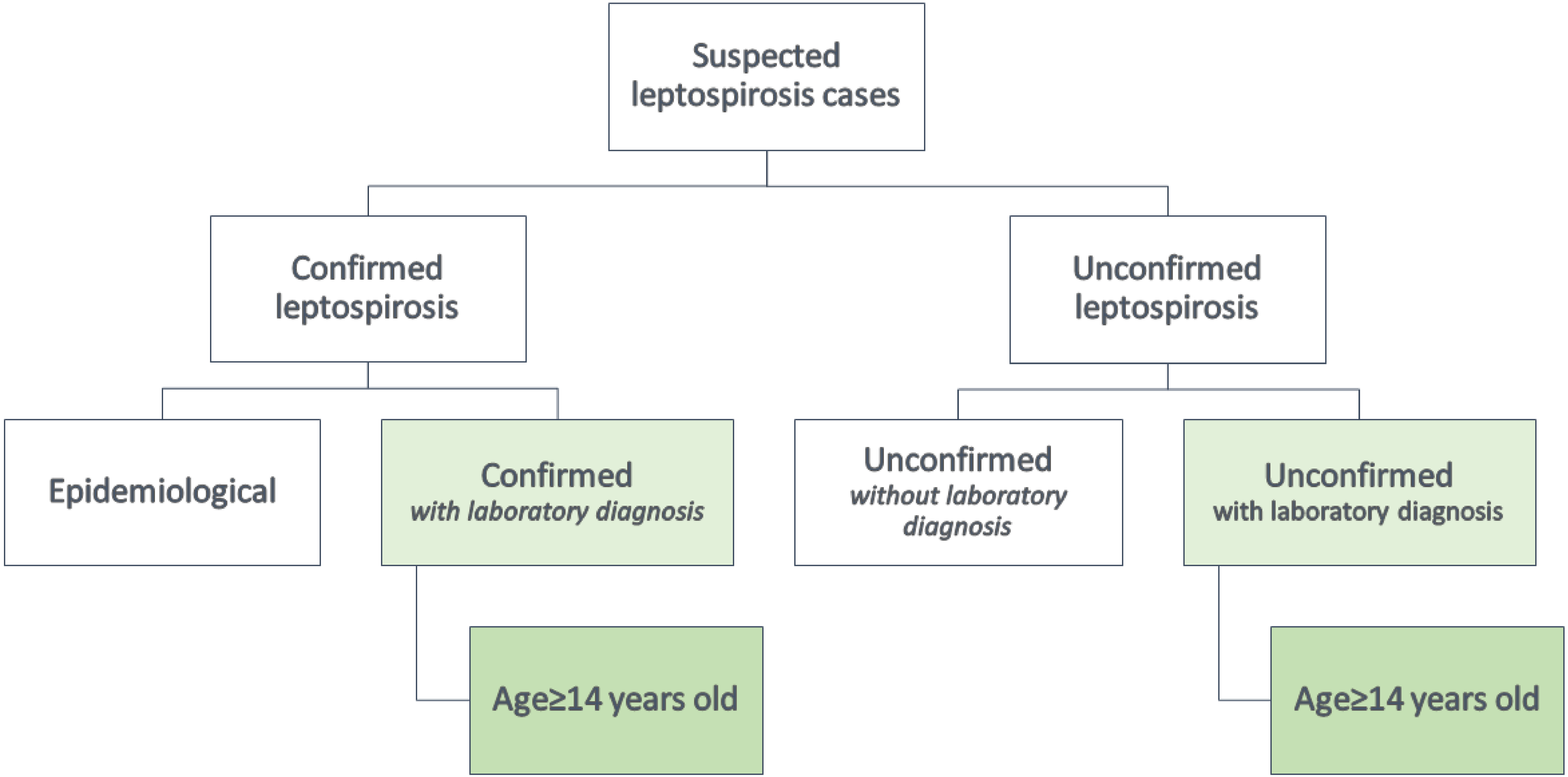

Suspected leptospirosis cases
Confirmed leptospirosis
Unconfirmed leptospirosis
Epidemiological
Confirmed
with laboratory diagnosis
Unconfirmed
without laboratory diagnosis
Unconfirmed
with laboratory diagnosis
Age≥14 years old
Age≥14 years old
